# Dataset of international students’ acceptance of online distance learning during COVID-19 pandemic: A preliminary investigation

**DOI:** 10.1016/j.dib.2022.108232

**Published:** 2022-05-01

**Authors:** Steve Shi-Hui, Lee Yen Chaw, Eugene Cheng-Xi Aw, Rohana Sham

**Affiliations:** a UCSI Graduate Business School, UCSI University, No. 1, Jalan Menara Gading, UCSI Heights (Taman Connaught), Cheras, Kuala Lumpur 56000, Malaysia; b Faculty of Business and Management, UCSI University, No. 1, Jalan Menara Gading, UCSI Heights (Taman Connaught), Cheras, Kuala Lumpur 56000, Malaysia

**Keywords:** Online learning, E-learning, Online education, UTAUT model, Institutions of higher learning (IHLs), Malaysia

## Abstract

The dataset describes factors affecting international students’ acceptance of Online Distance Learning (ODL) mode while pursuing oversea education during COVID-19 pandemic. The recruited respondents comprised of international students who were pursuing undergraduate degree programmes in the institutions of higher learning (IHLs) in Malaysia. Respondents were invited to participate in an online survey via Google Forms. A purposive sampling technique was adopted in this research whereby a total of 207 valid questionnaires were obtained and used for data analysis. Data outputs such as respondents’ profile, Partial Least Squares Structural Equation Modelling, and importance-performance matrix analysis were presented. The data can be used as a reference source to identify areas of improvement by educators, academic management, and policy makers of IHLs.

## Specifications Table

**Table:** 

Subject	Education
Specific subject area	Online learning
Type of data	Table and Figure
How the data were acquired	Data was collected using Google Forms, an online survey platform. The questionnaire is provided as a supplementary document.
Data format	Raw. analysed. Filtered. Descriptive and inferential statistics.
Description of data collection	Data were collected from international students of five randomly selected universities in Malaysia namely UCSI University, Taylor's University, Asia Pacific University of Technology & Innovation (APU), University of Nottingham Malaysia and University Science Malaysia (USM) using the purposive sampling technique. Before the survey link was disseminated to the international students, the researchers had obtained prior consensus from the School Representatives of the five universities for data collection. In the survey form, it was indicated that the respondents’ identity will remain anonymous and confidential. The final sample size consisted of 207 valid responses.
Data source location	Data were collected from four private universities which are located in Klang Valley of Malaysia and one public university from the northern region of Malaysia.
Data accessibility	All the data is attached with the article and in Mendeley Data: https://data.mendeley.com/datasets/9gbr7sjk32/1

## Value of the Data


 

• The data collected enable IHLs to identify vital factors that influence international students’ decision in accepting ODL mode for oversea education during COVID-19 pandemic.
• The data revealed areas of improvement in terms of teaching and learning mode that can be addressed by academic management or policy makers of the institutions.
• The dataset covers majority of the programmes offered by IHLs which can be used for further analysis.
• The dataset can be reused by educators or academic researchers who want to compare similar dataset as a preliminary investigation purpose.

## 1 Data Description

In this article, Online Distance Learning (ODL) is defined as a teaching method that is conducted online whereby instructors and students can interact by means of electronic channels and meetings [1,2]. Using a power level of 0.80, alpha value of 0.05 and effect size of 0.15, the minimum sample size generated by G*Power (version 3.1.9.4) was 85 samples. The final sample size of 207 obtained was more than the required threshold.

The data survey file was saved in Microsoft Excel spreadsheet accompanied this article which contained 207 rows and 24 columns. Each item was assigned a code as shown in Table 1. Items were measured by nominal, ordinal, or scale.

**Table 1:** Label of data.

Constructs	Items	Code	Measure
Gender	Male Female	1 2	Nominal Nominal

Age	Below 18 18–19 19–20 21 and above	1 2 3 4	Ordinal Ordinal Ordinal Ordinal

Programme Name	Business/Management	1	Ordinal
	IT/Computer Science Engineering/Architecture Education Hospitality/Tourism Performing Arts/Design Law Medicine/Nursing/Pharmacy Linguistics/Literature Applied Science Others	2 3 4 5 6 7 8 9 10 11	Ordinal Ordinal Ordinal Ordinal Ordinal Ordinal Ordinal Ordinal Ordinal Ordinal

Prior Experience	Yes	1	Nominal
	No	2	Nominal

Performance Expectancy	ODL is useful ODL gives me flexibility ODL fits my purpose	PE1 PE2 PE3	Scale Scale Scale

Effort Expectancy	ODL improves my learning ODL is clear ODL is easy to follow ODL is easy to master internet skills ODL easy to understand	PE4 EE1 EE2 EE3 EE4	Scale Scale Scale Scale Scale

Social Influence	Parents or guardians Friends or classmates Lecturers or professors My institution	SI1 SI2 SI3 SI4	Scale Scale Scale Scale

Facilitating Conditions	Necessary resources Necessary knowledge Technical support Academic support	FC1 FC2 FC3 FC4	Scale Scale Scale Scale

Acceptance behaviour	Use ODL for oversea education Use ODL if learning content Seriously thought of accepting ODL Plan to use ODL for future education	BI1 BI2 BI3 BI4	Scale Scale Scale Scale

Table 2 shows respondents’ profile. Of the 207 respondents, 49.8% were male students and 50.2% were female. Majority of the students were age 21 and above. Most students were from Business and Management as well as Engineering and Architecture programmes. 57.5% of respondents have prior experience with ODL whereas 42.5% do not.

**Table 2:** Respondents’ profile.

		Frequency	Valid Percent	Cumulative Percent
Gender	Male	103	49.8	49.8
	Female	104	50.2	100.0

Age	Below 18	26	12.6	12.6
	18–19	58	28.0	40.6

	19–20 21 and above	57 66	27.5 31.9	68.1 100.0

Programme name	Business/Management Engineering/Architecture Performing Arts/Art Design	46 39 21	22.2 18.8 10.2	22.2 41.0 51.2
	Applied Science Education IT/Computer Science Hospitality/Tourism Medicine/Nursing/Pharmacy Law Linguistics/Literature Others	13 12 12 9 9 8 7 31	6.3 5.8 5.8 4.3 4.3 3.9 3.4 15.0	57.4 63.2 69.0 73.3 77.6 81.5 84.9 100.0

Prior experience in using ODL	Yes No	119 88	57.5 42.5	57.5 100

In order to achieve the research's purposes, four core constructs, namely performance expectancy, effort expectancy, facilitating conditions and social influence are derived from the unified theory of acceptance and use of technology (UTAUT) model [3] to understand international students’ acceptance of ODL. Performance expectancy emphasizes on the expected benefits that can be provided by a system or technology. Effort expectancy is related to the easiness in using the system or technology. Facilitating conditions refers to the resources and support provided to perform a behaviour. Social influence indicates the extent to which users perceive their others such as peers or family members believe the technology to be important [3,4].

Based on the suggestion by Ringle and Sarstedt [5], Hair et al. [6] and Henseler et al. [7], the data were analysed using Partial Least Squares Structural Equation Modelling (PLS-SEM). Similar to Yuan et al. [8], Foo et al. [9], Tang and Chaw [10] and Aw et al. [15], a two-phases process was followed whereby the measurement model was assessed before the structural model. For measurement model assessment, the convergent validity and discriminant validity were evaluated. As shown in Table 3, the composite reliability (CR) and average variance extracted (AVE) values were above the thresholds of 0.70 and 0.50, respectively [6]. In addition, all item loadings were in the acceptable range between 0.633 and 0.871. Thus, it can be said that the convergent validity was achieved. Next, the discriminant validity was assessed using the heterotrait-monotrait ratio of correlations (HTMT) approach [7]. Table 4 showed that all HTMT values were below 0.90, indicating the establishment of discriminant validity.

**Table 3:** Measurement model.

Constructs	Indicators	Item loadings	CR	AVE
Performance expectancy	PE1	0.847	0.879	0.649
	PE2	0.633		
	PE3	0.868		
	PE4	0.850		

Effort expectancy	EE1	0.855	0.878	0.646
	EE2	0.854		
	EE3	0.648		
	EE4	0.839		

Social influence	SI1	0.816	0.844	0.578
	SI2	0.812		
	SI3	0.775		
	SI4	0.620		

Facilitating conditions	FC1	0.806	0.883	0.654
	FC2	0.858		
	FC3	0.802		
	FC4	0.767		

Acceptance behaviour	BI1	0.840	0.897	0.685
	BI2	0.793		
	BI3	0.871		
	BI4	0.804

**Table 4:** Discriminant validity.

	Effort expectancy	Facilitating condition	Acceptance behaviour	Performance expectancy	Social influence
Effort expectancy					
Facilitating conditions	0.738				
Acceptance behaviour	0.819	0.565			
Performance expectancy	0.881	0.758	0.786		
Social influence	0.754	0.778	0.733	0.754	

The structural model assessment began with the evaluation of variance inflation factor (VIF). The findings indicated that the VIFs were between 1.936 and 2.509, below the threshold of 3.3, implying no significant threat of multicollinearity in the dataset [6]. The R^2^ was 0.566, indicating 56.6% of variance in accepting ODL was explained by the proposed constructs. The model showed satisfactory model fit, with SRMR value (0.065) below the cut-off value of 0.08 [6]. Pertaining to path significance, as shown in Table 5, performance expectancy (*β* = 0.304, *p* < .05), effort expectancy (*β* = 0.363, *p* < .05), and social influence (*β* = 0.260, *p* < .05) showed significant positive effects on the ODL acceptance. However, the impact of facilitating conditions on ODL acceptance was not significant (*p* > .05).

**Table 5:** Relationships testing.

	Beta coefficient	T Statistics	*p*-value
Performance expectancy -> acceptance behaviour	0.304	3.795	0.000
Effort expectancy -> acceptance behaviour	0.363	4.310	0.000
Social influence -> acceptance behaviour	0.260	3.659	0.000
Facilitating conditions -> acceptance behaviour	−0.093	1.286	0.099

Finally, the Importance-Performance Matrix Analysis (IPMA) introduced by Ringle and Sarstedt [5] was performed to check the total effect and performance of proposed constructs (i.e., performance expectancy, effort expectancy, social influence and facilitating conditions). IPMA provided insights into the variables which were important but showed poor performance, thereby contributing to further managerial attention [11].

**Fig. 1:**
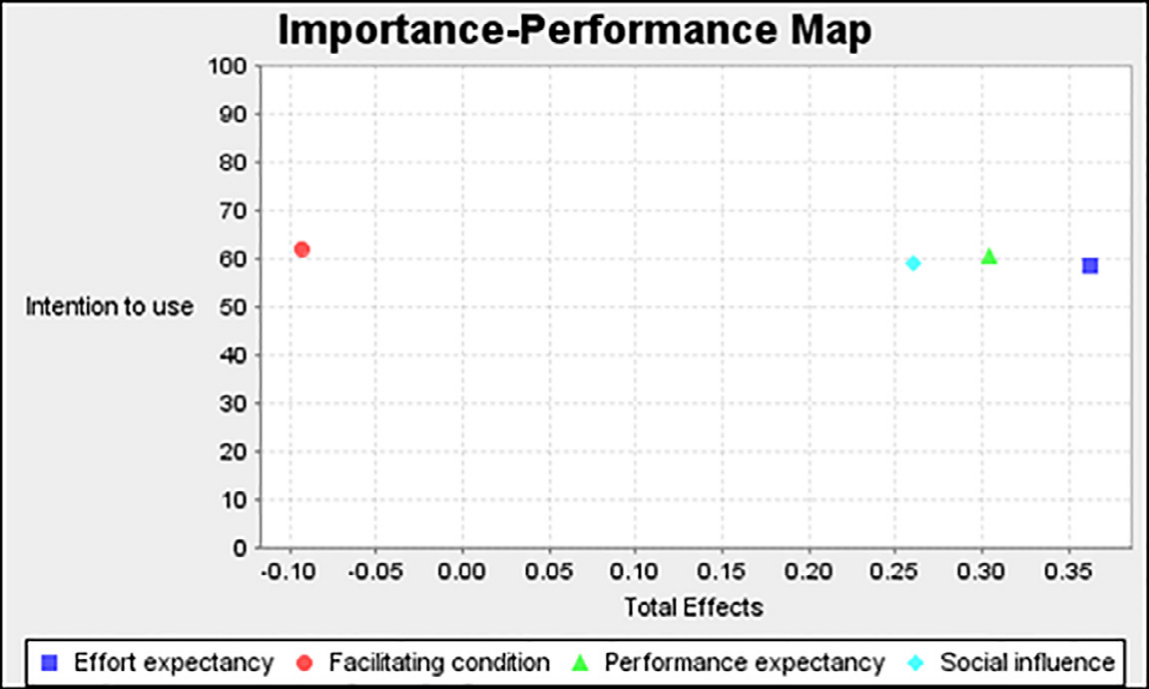
Importance-performance matrix analysis for accepting online distance learning mode.

As exhibited in Table 6 and Fig. 1, the factor with highest importance is effort expectancy (0.363) and the factor with least importance is facilitating conditions (−0.093). In terms of performance, facilitating conditions (62.096) topped the list, followed by performance expectancy (60.580), social influence (58.997), and effort expectancy (58.411). In sum, the IPMA analysis pointed out that effort expectancy could be of managerial importance, given that it is the most important construct in explaining acceptance of ODL, yet underperformed by the practitioners. On one hand, practitioners seem to overkill on the least important construct, namely facilitating conditions.

**Table 6:** Importance-performance matrix analysis.

	Importance	Performance
Performance expectancy	0.304	60.580
Effort expectancy	0.363	58.411
Social influence	0.260	58.997
Facilitating conditions	−0.093	62.096

## 2 Experimental Design, Materials and Methods

### 2.1 Questionnaire Design

A survey approached was adopted to gain insightful information with regard to international students’ intention to accept ODL mode for oversea education, particularly during COVID-19 pandemic period. The questionnaire consisted of two major parts. The first part included individual demographic characteristics such as gender, age, programme name and prior experience in using ODL. The second part of the questionnaire is related to factors affecting behaviour of international students’ acceptance of ODL mode for oversea education. The items used to measure the constructs (i.e. performance expectancy, effort expectancy, social influence, facilitating conditions and acceptance behaviour) were derived from previous studies [3,4,12] to ensure content validity. Additionally, a pre-test was carried out with three academic experts in this area. With their feedback, minor modifications were made on the questions and questionnaire layout. A 5-point Likert scale ranged from “1” (strongly disagree) to “5” (strongly agree) was employed to measure each of the main constructs in the questionnaire. Each of the constructs has 4 items, thus a total of 20 items appeared in the questionnaire.

### 2.2 Data Collection

The data were collected from international students of five randomly selected universities in Malaysia namely UCSI University, Taylor's University, Asia Pacific University of Technology & Innovation (APU), University of Nottingham Malaysia and University Science Malaysia (USM). Before the survey link was disseminated to the international students, the researchers have obtained prior consensus from the School Representatives of the five universities for data collection. In the survey form, it was indicated that the respondents’ identity will remain anonymous and confidential. The total duration of the data collection lasted two months from June to August 2020. Due to the reason that the sampling frame is not available for researchers, non-probability sampling technique was adopted. The approach has been widely adopted in similar situations or contexts [13]. We chose purposive sampling technique as it is suitable in achieving the research's purposes [14].

A total of 270 questionnaires were received. After performing data cleaning in SPSS, 63 questionnaires were discarded because they were not properly completed and suffered from straight-lining issue, leaving a total usable response of 207 for further analysis.

## Ethics Statements

Given that the research is a non-experimental voluntary survey, no ethical approval is necessary. Nevertheless, the consent of respondents to participate in the survey was still acquired beforehand, in an anonymous manner.

## CRediT authorship contribution statement

**Steve Shi-Hui:** Conceptualization, Methodology, Investigation, Writing – original draft. **Lee Yen Chaw:** Writing – original draft, Writing – review & editing, Visualization. **Eugene Cheng-Xi Aw:** Writing – review & editing, Formal analysis, Visualization. **Rohana Sham:** Writing – review & editing.

## Declaration of Competing Interest

The authors declare that they have no known competing financial interests or personal relationships that could have appeared to influence the work reported in this paper.

## Data Availability

Dataset related to Online Distance Learning (Original data) (Mendeley Data).

## Appendix Supplementary materials

Image, application 1
