# Supplementary material for: Dataset of international students’ acceptance of online distance learning during COVID-19 pandemic: A preliminary investigation
Source: Data Brief. 2022 May 1;42:108232. doi: 10.1016/j.dib.2022.108232 (PMC9057560; doi:10.1016/j.dib.2022.108232)
Supplement: Supplementary file 1 [file mmc1.docx]

**Dataset of international students’ acceptance of online distance learning during Covid-19 pandemic: A preliminary investigation**

**Survey Questionnaire**

This is an invitation for you to participate in a survey about your experience with online distance learning.

Please be assured that your responses will be completely anonymous. At no time will any individual be identified in any report because of this survey.

Thank you for your participation.

**Instructions:**

1. There are **two** (2) sections in this questionnaire. Please answer **ALL** questions in **ALL** sections.
2. Completion of this form will take you approximately 10 minutes.

Section A: Respondent Details

*Please tick your answer.*

QA1: Gender: ❑ Female ❑ Male

QA2: Age: ❑ Below 18 ❑ 18-19 ❑ 19-20 ❑ 21 and above

QA3: Program name:

| - Business or Management |
| --- |
| - Information Technology or Computer Science |
| - Engineering or Architecture |
| - Education |
| - Hospitality or Tourism |
| - Performing Arts or Art Design |
| - Law |
| - Medicine, Nursing or Pharmacy - Linguistics or Literature |
| - Applied Science |
| - Others: |

QA4: Do you have prior experience in using online distance learning? ❑ Yes ❑ No

Section B: The acceptance of Online Distance Learning for studying abroad

*This section is seeking your opinion regarding the factors that influence your acceptance towards online distance learning.*

*Respondents are asked to indicate the extent to which they agreed or disagreed with each statement using 5 Likert scale* [(1) = strongly disagree; (2) = disagree; (3) = neutral; (4) = agree; (5) = strongly agree; *Please circle one number per line to indicate the extent to which you agree or disagree with the following statements.*

| **No.** | **Items** | **Strongly Disagree** | **Disagree** | **Neutral** | **Agree** | **Strongly Agree** |
| --- | --- | --- | --- | --- | --- | --- |
| B1 | Performance Expectancy (PE) |  |  |  |  |  |
| PE1 | Online Distance Learning is useful to me. | 1 | 2 | 3 | 4 | 5 |
| PE2 | Online Distance Learning provides me with flexibility. | 1 | 2 | 3 | 4 | 5 |
| PE3 | Online Distance Learning fits my purpose of getting quality education. | 1 | 2 | 3 | 4 | 5 |
| PE4 | Online Distance Learning enables me to improve my learning. | 1 | 2 | 3 | 4 | 5 |
| B2 | Effort Expectancy (EE) |  |  |  |  |  |
| EE1 | Online Distance Learning is clear and understandable. | 1 | 2 | 3 | 4 | 5 |
| EE2 | Online Distance Learning is easy to follow. | 1 | 2 | 3 | 4 | 5 |
| EE3 | Online Distance Learning is easy for me to master internet skills. | 1 | 2 | 3 | 4 | 5 |
| EE4 | Online Distance Learning is easy for me to understand the learning content. | 1 | 2 | 3 | 4 | 5 |
| B3 | Social Influence (SI) |  |  |  |  |  |
| SI1 | My parents or guardians think that I should accept Online Distance Learning. | 1 | 2 | 3 | 4 | 5 |
| SI2 | My friends or classmates accept Online Distance Learning, so I should accept it as well. | 1 | 2 | 3 | 4 | 5 |
| SI3 | My lecturers or professors think that I should accept Online Distance Learning. | 1 | 2 | 3 | 4 | 5 |
| SI4 | My institution has supported the use of Online Distance Learning | 1 | 2 | 3 | 4 | 5 |
| B4 | Facilitating Conditions (FC) |  |  |  |  |  |
| FC1 | I have necessary resources to use Online Distance Learning | 1 | 2 | 3 | 4 | 5 |
| FC2 | I have necessary knowledge to use Online Distance Learning | 1 | 2 | 3 | 4 | 5 |
| FC3 | I have internal or external technical support to use Online Distance Learning | 1 | 2 | 3 | 4 | 5 |
| FC4 | I have academic support when I face problem with Online Distance Learning | 1 | 2 | 3 | 4 | 5 |
| B5 | Acceptance Behavioural (BI) |  |  |  |  |  |
| BI1 | I would use Online Distance Learning for oversea education | 1 | 2 | 3 | 4 | 5 |
| BI2 | I would use Online Distance Learning if the learning content and learning method are interesting | 1 | 2 | 3 | 4 | 5 |
| BI3 | I am seriously thinking of accepting Online Distance Learning in the next 2 months | 1 | 2 | 3 | 4 | 5 |
| BI4 | I plan to use Online Distance Learning for my future oversea education | 1 | 2 | 3 | 4 | 5 |

**Thank you for your time**

**~ The End ~**
